# Effects of High Frequency Repetitive Transcranial Magnetic Stimulation (HF-rTMS) on Delay Discounting in Major Depressive Disorder: An Open-Label Uncontrolled Pilot Study

**DOI:** 10.3390/brainsci9090230

**Published:** 2019-09-11

**Authors:** Juliana Teti Mayer, Magali Nicolier, Grégory Tio, Stephane Mouchabac, Emmanuel Haffen, Djamila Bennabi

**Affiliations:** 1 Service de Psychiatrie de l’Adulte, Centre Hospitalier Universitaire de Besançon, 25030 Besançon CEDEX, France; 2 Laboratoire de Neurosciences Intégratives et Cliniques EA 481, Université de Bourgogne Franche-Comté, 19 rue Ambroise Paré, 25000 Besançon, France; 3 Centre d’Investigation Clinique, INSERM CIC 1431, Centre Hospitalier Universitaire de Besançon, 25030 Besançon CEDEX, France; 4 Service de Psychiatrie de l’Adulte et Psychologie Médicale, APHP, Sorbonne Université, UPMC, Hôpital Saint-Antoine, F-75012 Paris, France; 5 Centre Expert Dépression Résistante FondaMental, Centre Hospitalier Universitaire de Besançon, 25030 Besançon CEDEX, France

**Keywords:** repetitive transcranial magnetic stimulation, high frequency repetitive transcranial magnetic stimulation, impulsivity, delay discounting, major depressive disorder

## Abstract

Background: Delay discounting (DD) refers to the decrease of a present subjective value of a future reward as the delay of its delivery increases. Major depressive disorder (MDD), besides core emotional and physical symptoms, involves difficulties in reward processing. Depressed patients often display greater temporal discounting rates than healthy subjects. Repetitive transcranial magnetic stimulation (rTMS) is a non-invasive brain stimulation technique applied in several countries to adult patients with treatment resistant depression. Studies suggest that this technique can be used to modulate DD, but no trial has assessed its effects on depressed patients. Methods: In this open-label uncontrolled trial, 20 patients diagnosed with MDD and at least stage II treatment resistance criteria underwent 20 HF-rTMS sessions over the dorsolateral prefrontal cortex (dlPFC; 10 Hz, 110% MT, 20 min). Pre-post treatment DD rates were compared. Effects on impulsivity, personality factors, and depressive symptoms were also evaluated. Results: No significant effect of HF-rTMS over the left dlPFC on DD of depressed individuals was observed, although rates seemed to increase after sessions. However, treatment resulted in significant improvement on cognitive impulsivity and depressive symptoms, and was well-tolerated. Conclusion: Despite the limitations involved, this pilot study allows preliminary evaluation of HF-rTMS effects on DD in MDD, providing substrate for further research.

## 1. Introduction

Major depressive disorder (MDD) is a widespread psychiatric disease that will become the leading worldwide cause of years living with disability by 2030 according to the World Health Organization (WHO). Episodes of depression, in addition to core emotional and physical symptoms, also involve difficulties in reward processing [1]. A reduction of reward anticipation, along with diminishment in anticipation predicting reduced motivation for rewards, has been observed in the acute phase [2]. Moreover, patients suffering from depression often display greater temporal discounting effects than healthy subjects. Depressed subjects highly value immediate rewards despite being offered a larger, albeit relatively delayed reward, which suggests changes on reward valuation [2,3,4]. This pattern has been related to anhedonia, which is a strong mediator for functional remission of depression [5] and could be predictive of suicide attempt lethality among depressed individuals [6,7].

Delay discounting (DD) or temporal discounting are terms that refer to the decrease of a present subjective value of a future reward as the delay of its delivery increases [8]. The degree of discount apparently has a trait-like characteristic, usually remaining stable for weeks, months, or even years [9]. It may be influenced, for example, by age, gender, culture, personality, and individual characteristics of sensation seeking (e.g., impulsivity or reward dependency) [9,10,11]. Higher scores on personality trait “neuroticism” have been positively associated to higher discount rates and thus higher devaluation of future rewards, while higher “consciousness” scores have exhibited a negative association, with lower devaluation of future rewards [10]. Furthermore, the DD paradigm has an important role in understanding impulsive behavior [9,12,13,14]. In the context of an intertemporal choice, the temporal impulsivity model corresponds to the preference for smaller, sooner rewards over larger, later ones, with steeper discount rates [8].

Tendency for impulsive choices and inability to delay gratification can stand in the way of one’s fulfillment of long-term plans. Individuals have different discount rates, and the more one devaluates a future reward as a function of its delay, the less influence this reward will have on one’s present choices [8]. In an evolutionary perspective, preferring immediate gratifications was associated with a strong conservative value due to the competition between individuals or other species over resources [15,16]. However, in our modern world, this assumption is not systematically valid. As we are often confronted with intertemporal decisions, studies concerning DD in the context of intertemporal decision-making are of high importance, since it impacts an individual’s education, health, and finances [9].

The behavioral balance between cautious and risky decisions critically involves the prefrontal cortex (PFC). This cortical area has been implicated in decision-making processes based on reward values and effort calculations of multiple choices to promote goal-directed behavior [9,17]. Activation of different brain areas has been observed in association with preference for either immediate or delayed rewards. According to McClure and colleagues [14], during impulsive choices, limbic and paralimbic areas (such as the ventral striatum, the medial orbitofrontal cortex, and the medial PFC) were significantly activated. These areas are known to be richly innervated by the dopaminergic system. On the other hand, preference for delayed rewards were related to a more preponderant activity of lateral prefrontal and associated parietal areas, such as the dorsolateral part of the PFC (dlPFC) [14].

Evidence suggests that non-invasive brain stimulation over the dlPFC can improve risk taking, reward seeking, computation of values, as well as other processes involved in impulsive behavior [18]. Transcranial magnetic stimulation (TMS) is a non-invasive technique based on the induction of an electric field in the brain by electromagnetic pulses. When pulses are delivered repetitively, the technique is called repetitive TMS (rTMS). This technique is applied in several countries to the treatment of MDD in adult patients who did not satisfactorily improve from antidepressant medication in the present episode, with the dlPFC as the main target [19,20]. Additionally, its application over the dlPFC is likely to modulate plasticity of the relevant cortico-subcortical network including cortical (e.g., other frontal regions such as the orbitofrontal/ventromedial) and subcortical structures, with potential to induce behavioral changes that can last beyond the time of stimulation [21,22].

Modulatory effects of TMS on DD are promising, as suggested by a recent review and meta-analysis on studies involving healthy adult participants, with a significant mean effect size for the left dlPFC as the target [23]. Another trial, not included in this review, obtained consistent findings: Figner and collaborators [24] observed that transient disruption of the left dlPFC with low-frequency rTMS increased DD rates of 55 healthy volunteers (15 min of 1 Hz rTMS, 54% of maximum stimulator output, 900 pulses).

Studies assessing TMS effects on the DD of clinical populations remain scarce. Sheffer and colleagues [25] delivered 900 pulses at either 10 or 20 Hz over the left dlPFC, with a 110% motor threshold (MT), in a population of 66 smokers and non-smokers. Discounting rates decreased and effects were more intense as frequency was higher. Recently, Sheffer and colleagues [26] demonstrated improvement on discount rates in a population of 29 tobacco smokers after 8 sessions of 20 Hz (900 pulses per sessions, 110% MT) also over the left dlPFC. Nonetheless, in another study by Zack and colleagues [27], no significant effect on nine men suffering from pathological gambling was reported after a single rTMS session (10 Hz, 80% AMT, 450 pulses) over the mPFC. Post-rTMS effects actually headed towards the opposite direction and were highly variable [27].

As far as we know, no study has assessed rTMS effects on DD in depressed individuals. We have therefore conducted an open-label uncontrolled pilot study to evaluate whether rTMS applied to the dlPFC could reduce DD in MDD patients who have not shown satisfactory clinical progress with medication. We hypothesized that rTMS would decrease temporal impulsivity of these patients, along with the expected improvement on their depressive symptoms. The primary objective was to analyze changes in baseline DD rates after 20 rTMS sessions. As secondary outcome measures, we also investigated (i) the neuromodulatory effects of a single rTMS session on baseline DD scores; (ii) the effects of 20 rTMS sessions on general impulsivity, (iii) on personality traits, and (iv) on depressive symptoms; (v) possible correlations of DD rates with the other psychiatric measures; and (vi) DD rates correlations with sociodemographic characteristics.

## 2. Materials and Methods

### 2.1. Participants

The study was conducted at the psychiatric department of the University Hospital of Besancon, France, and aimed to enroll all eligible volunteers that met inclusion criteria. The inclusion criteria were as follows: (i) men and women over 18 years old, with (ii) diagnosis of MDD according to the Diagnostic and Statistical Manual of Mental Disorders (DSM-IV) criteria, and (iii) at least stage II treatment resistant criteria [28,29]. Exclusion criteria were as follows: (i) age less than 18 years old, (ii) contraindication for brain magnetic resonance imaging (MRI) or TMS, (iii) presence of psychotic features and/or (iv) neurological comorbidity. Semi-structured psychiatric interviews to establish MDD diagnosis according to DSM-IV criteria and stage of treatment resistance were conducted by trained psychiatrists (E.H. or D.B.). Concomitant use of psychiatric medication other than antidepressants did not interfere with participation in the study. Twenty patients were included and submitted to brain MRI prior to rTMS sessions.

### 2.2. Study Design

In this open-label uncontrolled trial, all subjects underwent the same procedure. Patients received 20 sessions of high frequency rTMS (HF-rTMS), delivered twice a day over a period of two weeks (sessions were performed by E.H. or D.B. from Monday to Friday). The protocol was approved by the French Committee of Protection of Persons (CCPPRB Reference No.: 04/380) and authorization was given by the General Health Administration (DGS 2005/0030). Investigations were conducted in line with the principles from the Declaration of Helsinki. Signed written informed consent was obtained from all patients before study enrollment.

#### 2.2.1. Intervention

A Magstim Super Rapid2 (Magstim Company Ltd., Whitland, Wales, UK) with an air-cooling figure-of-eight coil was used. The rTMS was administered at 10 Hz during 5 s, with 25 s between trains, and at 110% MT over the left dlPFC per 20 min session (2000 stimuli per day). The coil was angled tangentially to the head. The placement to stimulate the left dlPFC was 5 cm anterior to the hand motor area, in a parasagittal line. Tolerability and side effects were assessed both verbally, during and after sessions, and through a Visual Analogue Scale (VAS) applied by the end of sessions. The VAS assessed the patient’s perception on pain, mood, fatigue, and motivation.

#### 2.2.2. Outcome Measures

Measures on DD rates were collected at three points in time: prior to stimulation sessions (baseline scores), immediately after the first session, and after 20 sessions. DD was assessed by the 27-item Monetary Choice Questionnaire (MCQ), based on Kirby’s DD inventory [8]. The MCQ is a pen-and-paper task composed of 27 items of hypothetical monetary choices between smaller immediate rewards and larger delayed ones. Items are classified into three categories: small, medium, or large rewards [8]. Monetary values were converted into local currency (EUR; €). Delays varied between seven and 186 days, and rewards ranged from 11€ to 85€.

Furthermore, measures of impulsivity, personality traits, and severity of depressive symptoms were collected prior to stimulation sessions and at the end of 20 rTMS sessions. Impulsivity was evaluated by the French version of the Barratt Impulsiveness Scale (BIS-10) [30,31], a 34-item self-rated checklist that gives overall and specific impulsivity scores based on three subscales: cognitive-, motor-, and non-planning-impulsivity. Assessment of major personality traits was obtained through the French version of the Big Five Inventory (BFI-Fr) [32]. This self-rated questionnaire independently evaluates the following personality factors: conscientiousness, neuroticism, extraversion, openness to experience, and agreeableness.

Depression severity was assessed by both the Montgomery–Åsberg Depression Rating Scale (MADRS) [28]—filled by a trained psychiatrist—and by the self-rated version of the Quick Inventory of Depressive Symptomatology (QIDS-SR16) [33]. The MADRS is a ten-item clinician-rated scale, with a score ranging from 0 to 60 (6 points per item). Cutoff points are 0–6 asymptomatic; 7–19 mild depression; 20–34 moderate depression; and >34 severe depression. The QIDS-SR16 has 16 items (score range from 0 to 27) assessing the severity of depressive symptoms as perceived by the subject, with the following cutoff points: 0–5 none; 6–10 mild; 11–15 moderate; 15–20 severe; and 21–27 very severe depression.

### 2.3. Data Analysis

DD rates are based on the hyperbolic function V = A/1 + *k*D, where V represents the subjective value of the delayed reward, A is the amount of the delayed reward, D is the delay, and *k* is the coefficient that estimates the subjective discounting rate for the given delayed reward. The hyperbolic function is more efficient than the exponential model of discounting, since it better integrates discount variations for decisions concerning rewards in the short and in the long run [34]. *K*-values were generated by the 27-item MCQ Automated Scores [35] for small, medium, and large rewards, as well as for overall discounting rates of each subject. Given the small sample size, the Wilcoxon sign rank test (non-parametric test) was performed for all hypothesis testing. Spearman’s rho was used to explore correlations. The alpha value for significance was set at 0.05. Bonferroni’s correction for significance was applied for multiple comparisons, with the significance level adapted for each explored objective (primary and secondary outcome measures). The ratings were analyzed with SAS 9.4 T5 Level MM3.

## 3. Results

### 3.1. Participants Characteristics, Missing Data, and Outliers

Our sample was composed of 9 women (45%) and 11 men (55%), with a mean age of 54.4 (SD ± 9.854) years. All patients had been using antidepressant medications for at least 4 weeks: 18 of them were on antidepressant monotherapy (selective serotonin reuptake inhibitor (SSRI) = 7, serotonin and norepinephrine reuptake inhibitor (SNRI) = 7, tricyclic antidepressant (TCA) = 3, tetracyclic antidepressant (TeCA) = 1), and two patients had a combination of two antidepressants (SNRI with TeCA = 2). Mean baseline scores and demographic data are displayed on Table 1. Far outliers were removed based on the z-score method with a SD 3.29 (0.01% of data was removed). Additionally, data were missing completely at random for some participants. Hence, the number of subjects whose data were considered for analysis is detailed in the “Subjects (*n*)” column.

### 3.2. Impact of 20 rTMS Sessions on DD

**Figure 1:**
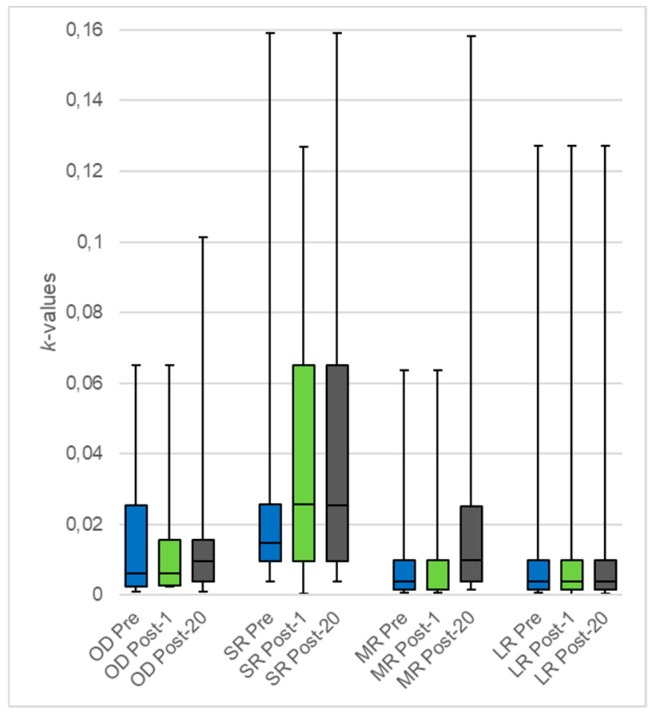
Boxplot for median *k*-values for the different reward categories pre-post rTMS sessions. OD: overall discounting, SR: small rewards, MR: medium rewards, LR: large rewards, Pre: pre-rTMS sessions/baseline measure (blue), Post-1: post-first rTMS session (green), Post-20: post-20 rTMS sessions (grey).

The effect on small, medium, large rewards, and overall discount rates (four comparisons; *p* < 0.0125) was assessed. The median values apparently increased after rTMS sessions (Figure 1), especially for medium rewards (*p* = 0.0293). However, analysis showed that there was no statistical significance (Table 2).

### 3.3. Impact of One rTMS Session on DD

As a secondary outcome, the effect of a single rTMS session on the same variables was also analyzed (*p* < 0.0125), but no significant change was observed (Figure 1).

### 3.4. Impact of rTMS on the BIS-10

The analysis of the impact of 20 rTMS sessions on the BIS-10 considered four scores: BIS-10 total score and cognitive-, motor-, and non-planning-impulsivity subscales scores (*p* < 0.0125). There was a significant reduction on cognitive-impulsivity scores (*p* = 0.0071) as displayed in Table 2. No significant change was observed in the other scores.

### 3.5. Impact of rTMS on the BFI-Fr

No significant effect of 20 rTMS sessions on the five personality traits assessed by the BFI-Fr (*p* < 0.01) was observed (Table 2).

### 3.6. Impact of rTMS on Depressive Symptoms

Depressive symptoms assessed by the MADRS and the QIDS-SR16 (*p* < 0.025) were significantly reduced in both assessments (*p* = 0.0234; *p* = 0.0240, respectively) at the end of 20 rTMS sessions (Table 2).

### 3.7. Correlation between DD and Other Psychiatric Measures

Spearman’s rho test revealed significant correlations (*p* < 0.05) between pre-post 20-rTMS-session discounting rates and scores of other psychiatric measures. The agreeableness score on the BFI-Fr was negatively correlated to *k*-values for medium rewards (rho = −0.62658; *p* = 0.0071), while the non-planning-impulsivity subscale from the BIS-10 was positively correlated to *k*-values for large rewards (rho = 0.49898; *p* = 0.0491). No other significant correlations were found. Baseline psychiatric scores, age, and gender of participants had equally no significant influence on pre-post 20 rTMS discount rates.

### 3.8. Side Effects

All treatment sessions were well tolerated and subjects reported no side effects.

## 4. Discussion

The present study found no significant effect of 20 HF-rTMS over the left dlPFC on temporal impulsivity of patients diagnosed with MDD and meeting at least stage II criteria for treatment resistance. We expected that rTMS sessions would improve temporal discount rates in patients with MDD, based on previous studies with healthy participants. However, our results did not support this simple hypothesis. However, treatment significantly improved cognitive-impulsivity scores and depressive symptoms with no side effects reported.

There are limitations of this trial that must be acknowledged before further discussion. A major limitation is our small sample size, which may compromise the generalizability of outcomes. In addition, confounding factors could influence the results due to the open-label uncontrolled study design and potentially compromise internal validity. On the other hand, these preliminary findings might be useful for directing further research in the field because our study is strengthened by well-defined eligibility criteria for the investigated population, outcome assessment by validated instruments only, and the adjustment of confidence intervals for multiple comparisons.

Interestingly, as we analyzed data from pre-post 20 HF-rTMS sessions, an apparent increase on temporal impulsivity—especially for medium rewards (*p* = 0.0293)—was observed, although no significance of the effect was obtained. Methodological differences between our trial and previous trials with healthy participants [23] make direct comparison of results unfeasible, whereas data on the assessment of clinical populations remain insufficient. Factors that may influence rTMS efficacy such as the cortical target, stimulation parameters, and inter-individual variations require re-examination in light of previous evidence.

The choice of cortical target and stimulation parameters were in line with prior evidence regarding rTMS application on depression [19,20,36]. When it comes to stimulation parameters, neuromodulation by rTMS has shown therapeutic effects and is usually performed at 10 Hz when applied over the left dlPFC of depressed individuals, with an increase in responsiveness observed when the number of sessions is greater than 10 [19]. The chosen parameters in our study have shown themselves to be effective on this population since expected improvements on secondary outcomes were observed.

An important aspect to be considered while evaluating neuromodulatory results is the influence of inter-individual features. A large variability of outcomes is frequently reported by non-invasive modulation experiments and has sometimes been associated with characteristics such as age, gender, baseline cortical activation, brain metabolic activity, neural tissue heterogeneity, and even smoking status [37,38,39,40,41,42].

MDD is frequently associated with changes in brain functionality and structure [43,44], such as progressive neurodegeneration in the left dlPFC [45]. Pathological regions may promote changes in TMS-induced currents, modifying the magnitude, location, and orientation of current distribution, due to local perturbation in conductivity [46]. Drysdale and colleagues [47] have recently observed four depression biotypes in a cluster analysis of resting-state functional MRI (fMRI) data. Although the biotypes shared a common neuroanatomical pathological core, they displayed different abnormal connectivity patterns, especially regarding frontoamygdala, thalamic, and frontostriatal networks, as well as anterior cingulate and orbitofrontal areas. The biotypes were also associated with different a responsiveness to rTMS when applied to the dorsomedial PFC (dmPFC) [47].

Moreover, trials merging TMS and brain-imaging methods have demonstrated that HF-rTMS can induce changes on dopamine release in the striatum [48,49,50,51,52]. In the context of MDD, Pogarelli and colleagues [52] described an induction of endogenous dopamine release on single photon emission computed tomography (SPECT) of patients with moderate to severe depression, as a result of a three week HF-rTMS standard treatment over the left dlPFC. Considering these findings, the rTMS impact on DD behavior of depressed individuals needs to be carefully assessed by further studies, since relation between increased striatal dopaminergic activity and immediacy has been previously described [12,14].

As a secondary outcome, we have detected a significant decrease in baseline cognitive impulsivity. This result is in accordance with previous findings on the promising effect of neuromodulation on impulsive behavior [18,23]. Stimulation sessions also showed a significant effect on MDD symptomatology. Clinical improvement was both perceived by the specialist (MADRS) and reported by the patients (QIDS-SR16). This effect is in accordance with solid literature on the field [19,20].

Correlation analysis revealed that the more the participants scored on the non-planning-impulsivity subscale, the steeper they discounted on large delayed rewards. A noteworthy observation is that not all BIS-10 subscales were correlated to DD behavior, and the significant improvement on the cognitive-impulsivity subscale occurred independently from other impulsivity dimensions. These results reinforce the hypothesis of impulsive behavior as a multifaceted complex phenomenon [53,54], and that tools employed to assess its dimensions probably reflect separate underlying processes, which results in a general lack of intercorrelation [13,17,55,56]. Regarding the BFI-Fr personality factors, data suggested that the less the subjects scored on characteristics of agreeableness, the steeper they discounted on medium delayed rewards. This negative correlation is in accordance with previous findings [57], albeit little attention has been given in the literature to the relation between these variables.

Since high discount rates may interfere with long-term plan fulfillment and quality of life, further investigation is necessary to clarify the effect on DD associated with rTMS application in MDD. Randomized controlled trials with larger population samples should be performed in order to strengthen internal and external validity. In addition, comparison between online and offline scores and/or association with brain imaging techniques (SPECT, fMRI) may lead to a better understanding on rTMS effects. Other targets could also be explored in accordance with depression biotypes and their particular connectivity dysfunction [47] as therapeutic alternatives for both depression and impulsivity. Lastly, given the complex nature of impulsivity, researchers could consider combining instruments to simultaneously measure other impulsive dimensions (such as response inhibition, planning) and obtain a more comprehensive assessment of the phenomenon.

## 5. Conclusions

Despite the limitations involved in this study, the results allow a preliminary evaluation of rTMS effect on temporal impulsivity in depressed individuals meeting treatment-resistant criteria. Nevertheless, further research concerning MDD and DD is required. Merging TMS with brain-imaging methods, performing randomized controlled trials, and associating measure instruments on impulsivity may help us better understand the effect of neuromodulation on temporal impulsivity in depression.

## Figures and Tables

**Table 1:** Mean scores for quantitative population data and baseline measures.

Measure	Subjects (*n*)	Mean	SD
Age	20	54.40	9.85
MCQ (*k*-value)			
Overall	17	0.017	0.021
Small Rewards	18	0.030	0.039
Medium Rewards	18	0.012	0.019
Large Rewards	17	0.014	0.030
MADRS	11	28.64	5.33
QIDS-SR16	18	23.61	8.10
BIS-10			
Overall	16	74.81	20.36
Cognitive-impulsivity	16	29.37	6.04
Motor-impulsivity	16	21.31	6.91
Non-planning-impulsivity	16	24.56	9.24
BFI-Fr			
Conscientiousness	18	28.22	7.34
Neuroticism	18	29.28	9.20
Extraversion	18	18.11	5.70
Openness to Experience	18	25.89	5.27
Agreeableness	18	41.50	6.16

MCQ: 27-item Monetary Choice Questionnaire, MADRS: Montgomery–Åsberg Depression Rating Scale, QIDS-SR16: self-rated version of the Quick Inventory of Depressive Symptomatology, BIS-10: Barratt Impulsiveness Scale-10, BFI-Fr: Big Five Inventory (French version), SD: standard deviation.

**Table 2:** Median values and quartiles (Q1;Q3) for pre-post 20 rTMS sessions measures with *p*-values.

	Subjects (*n*)	Median (Q1;Q3)		*p*-Value
		Pre-rTMS	Post-20 Sessions	
MCQ (*k*-value)				
Overall	17	0.006 (0.002;0.025)	0.010 (0.004;0.016)	0.855
Small Rewards	18	0.015 (0.010;0.026)	0.025 (0.010;0.065)	0.147
Medium Rewards	18	0.004 (0.002;0.010)	0.010 (0.004;0.025)	0.029
Large Rewards	17	0.004 (0.002;0.010)	0.004 (0.002;0.010)	0.303
BIS-10				
Overall	16	76 (66;90.5)	73.5 (63.5;87.5)	0.131
Cognitive-impulsivity	16	31 (26;33)	29.5 (25;32)	0.007 *
Motor-impulsivity	16	20.5 (17;26.5)	20 (14.5;26.5)	0.293
Non-planning-impulsivity	16	26.5 (20.5;30.5)	25.5 (17.5;29.5)	0.577
BFI-Fr				
Conscientiousness	18	26.5 (23;34)	27.5 (26;35)	0.341
Neuroticism	18	32 (27;35)	33 (29;35)	0.585
Extraversion	18	18.5 (13;24)	15.5 (13;21)	0.061
Openness to Experience	18	25.5 (23;29)	24.5 (21;27)	0.297
Agreeableness	18	42 (38;45)	41 (36;44)	0.267
MADRS	11	27 (24;32)	21 (15;29)	0.023 *
QIDS-SR16	18	25.5 (20;28)	19 (14;24)	0.024 *

* Statistically significant *p*-values. MCQ: 27-item Monetary Choice Questionnaire, BIS-10: Barratt Impulsiveness Scale-10, BFI-Fr: Big Five Inventory (French version), MADRS: Montgomery–Åsberg Depression Rating Scale, QIDS-SR16: self-rated version of the Quick Inventory of Depressive Symptomatology.
